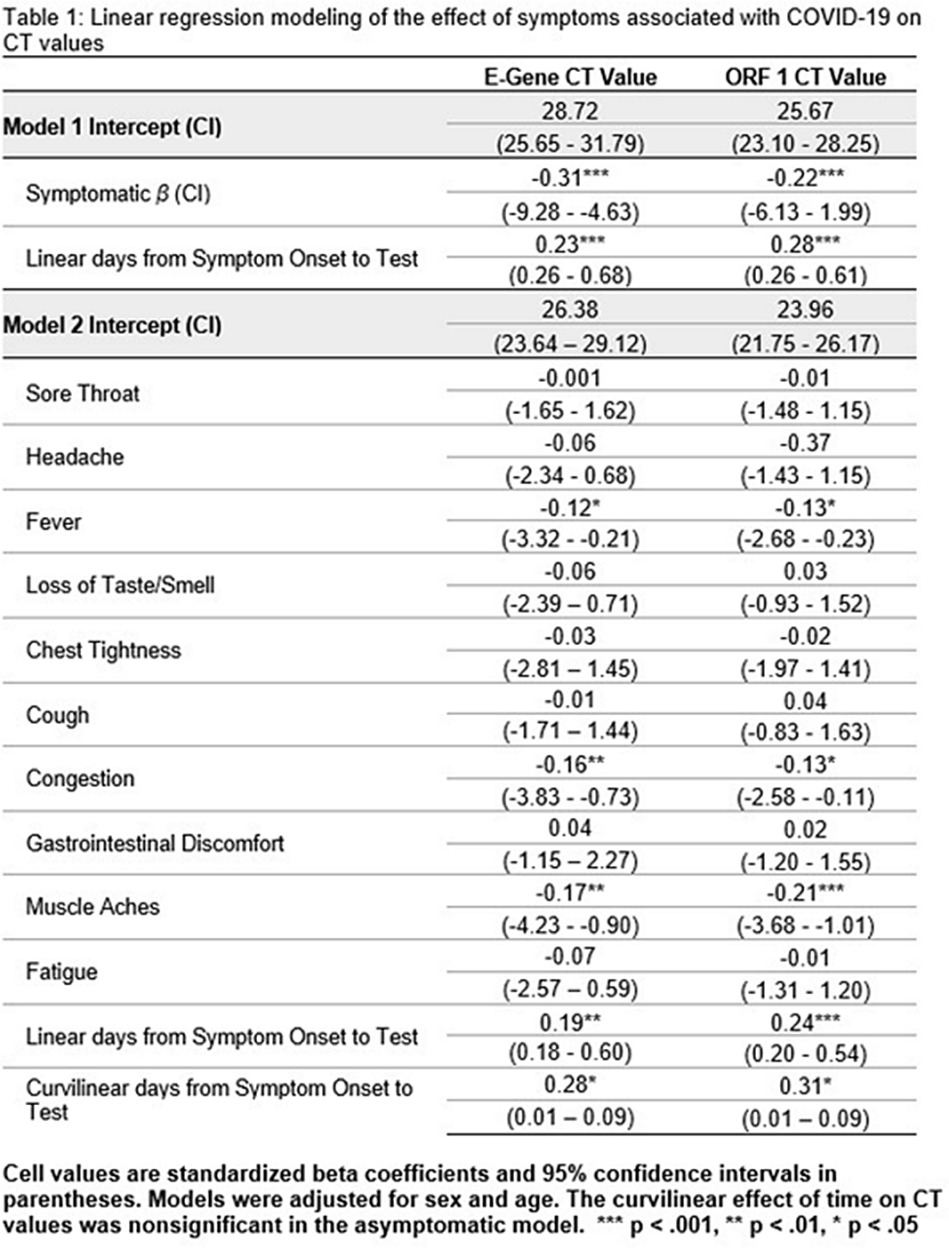# Evaluating the Relationship between Cycle Threshold Values and Reported COVID-19 Symptoms among Healthcare Workers

**DOI:** 10.1017/ash.2021.35

**Published:** 2021-07-29

**Authors:** Mindy Sampson, Catherine Passaretti, Jennifer Priem, Shelley Kester, Kristin Fischer, John Longshore

## Abstract

**Background:** SARS-CoV-2 detected by reverse transcription polymerase chain reaction (RT-PCR) can persist for weeks to months in some individuals. Cycle threshold (Ct) values represent the number of cycles needed to amplify viral ribonucleic acid (RNA) to reach a detectable level. As such, Ct values are inversely related to the amount of virus in a sample. As knowledge of SARS-CoV-2 viral dynamics continues to evolve, understanding the relationship between Ct values, type of symptoms, and timing of symptom onset can help determine when infected individuals are most likely to be infectious. **Methods:** We conducted a retrospective cohort study of 1,027 healthcare workers (HCWs) who tested positive for SARS-CoV-2 by RT-PCR from nasopharyngeal specimens between June 27, 2020, and September 21, 2020. All HCWs were interviewed within 72 hours of their diagnosis for symptom history. Due to multiple PCR platforms being in use in our facility, only 360 HCWs (35%) had Ct values available for analysis. Multivariate linear regression models examined the effect of COVID-19–related symptoms and timing of symptom onset to test on Ct values. **Results:** The most frequently reported symptoms were congestion (55.6%), cough (50.3%), and headache (46.7%). Other symptoms less commonly reported were fatigue (36.7%), loss of taste or smell (36.4%), fever (35.4%), muscle aches (33.3%), sore throat (27.4%), and diarrhea (26.7%). Symptomatic HCWs (88.3% of sample) had lower Ct values (ORF-1 M = 22.66, SD = 5.17; E-Gene M = 24.34, SD = 6.60) than asymptomatic individuals (ORF-1 M = 25.46, SD = 6.06; E-Gene M = 29.34, SD = 7.96). Of all symptoms measured, only presence of fever, congestion, and muscle aches predicted significantly lower Ct values. Mean Ct values decreased 2 days prior to symptom onset, were lowest the day of symptom onset, then increased in a curvilinear fashion. There were no significant 2-way interactions between symptoms and time of symptom onset to testing. **Conclusions:** The curvilinear pattern of Ct values over time from symptom onset are consistent with disease progression patterns and support current understanding of infectivity being highest 2 days prior to symptom onset through day 8. Presence of fever, congestion, and muscle aches are significantly correlated with lower Ct values, suggesting that these symptoms are associated with higher viral load. Although Ct values are not without limitations, our findings support the current understanding that presymptomatic and symptomatic individuals, particularly those with fever, congestion, and muscle aches, may pose higher risk of transmission to others.

**Funding:** No

**Disclosures:** None

**Figure 1.**
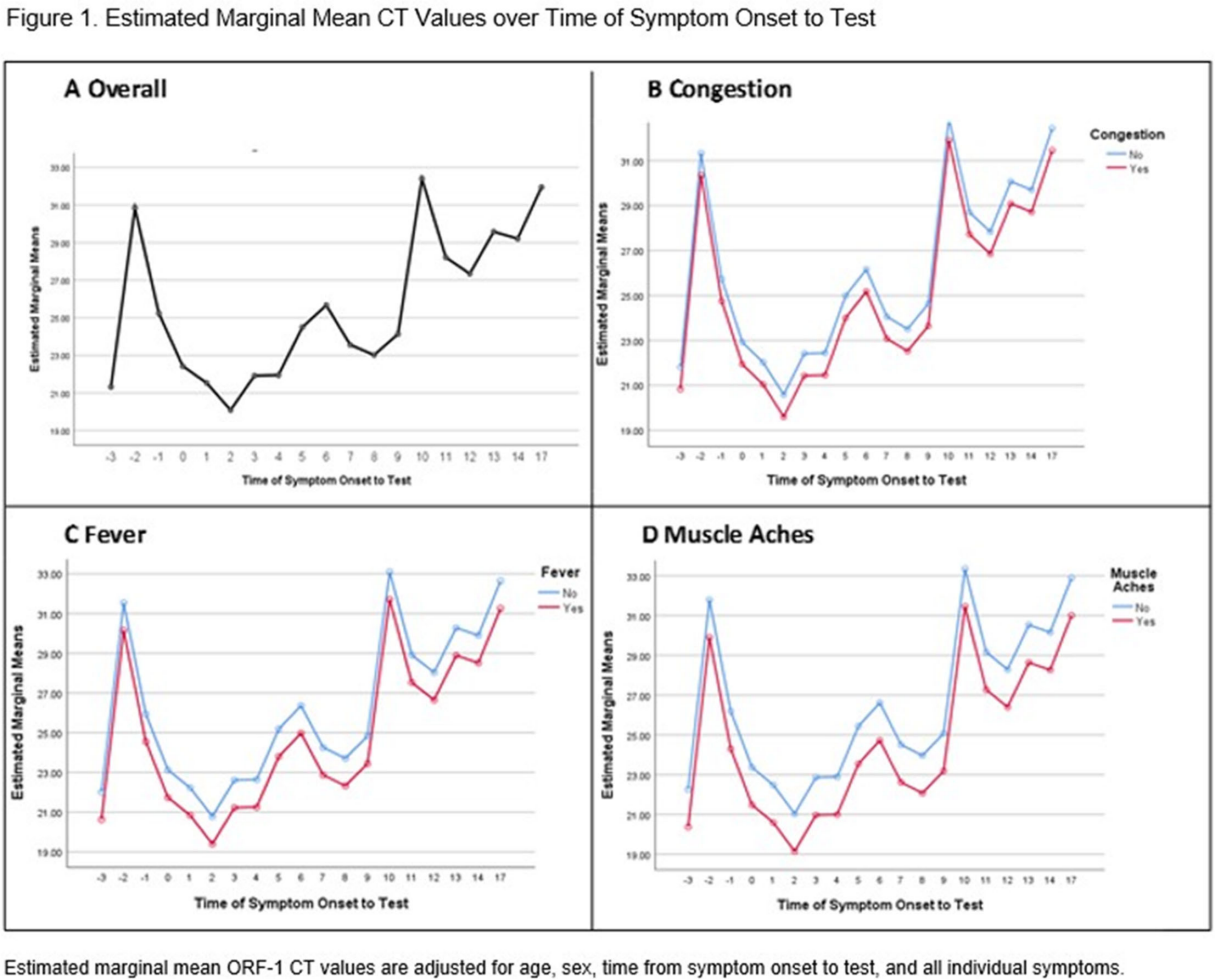

**Figure 2.**
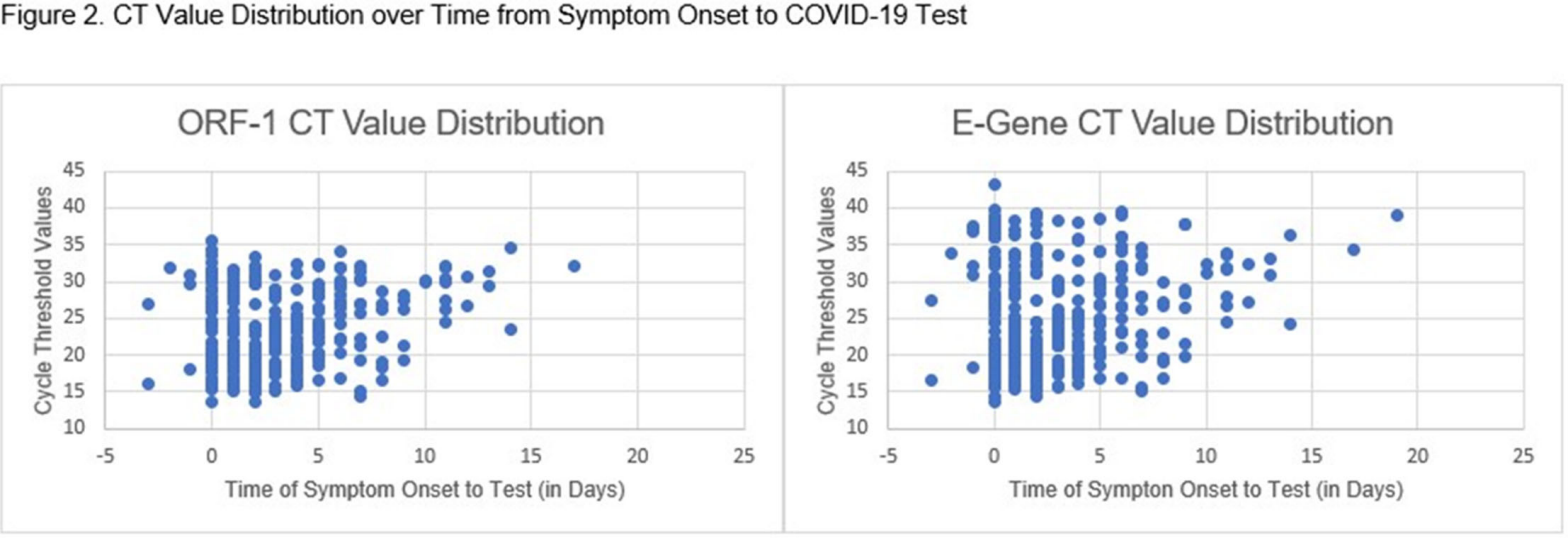

**Table 1.**